# Appropriate linear stapler selection for avoiding postoperative pancreatic fistula after distal pancreatectomy: A retrospective cohort study

**DOI:** 10.1097/MD.0000000000039954

**Published:** 2024-10-04

**Authors:** Kei Naito, Kazuyasu Shinmura, Takayuki Suzuki, Shintaro Maeda, Satoshi Kuboki, Masayuki Ohtsuka

**Affiliations:** a Department of Surgery, Saitama Red Cross Hospital, Japanese Red Cross Society, Saitama, Japan; b Department of General Surgery, Graduate School of Medicine, Chiba University, Chiba, Japan.

**Keywords:** distal pancreatectomy, linear stapler, postoperative pancreatic fistula

## Abstract

**Background:**

Despite the progress in surgical techniques and perioperative managements, the incidence of postoperative pancreatic fistula (POPF) after distal pancreatectomy (DP) remains high. Recently, pancreatic dissection using a linear stapler has been widely performed; however, risk factors influencing the occurrence of POPF after DP using a liner stapler is not fully understood. The purpose of this paper was to evaluate whether the relations between staple height and pancreatic thickness or main pancreatic duct (MPD) diameter influenced the incidence of POPF.

**Methods:**

Patients who underwent DP without other organ resections between 2015 and 2022 were retrospectively reviewed. Compression Index (CI) was defined as staple height/pancreatic thickness, and Suturing Index (SI) was defined as staple height/ MPD diameter.

**Results:**

In 51 patients undergoing DP, 16 patients (31.4%) developed POPF. ROC analyses revealed that lower CI and higher SI significantly increased the incidence of POPF, and the cutoff values were 0.186 and 0.821, respectively. Univariate and multivariate analyses revealed that CI ≤ 0.186 and SI ≥ 0.821 were independent risk factors for POPF after DP. Moreover, the incidence of POPF in patients fulfilling both CI > 0.186 and SI < 0.821 was 5.9%, which was extremely lower than in those without fulfilling the criteria (44.1%), suggesting that this new criteria in combination with CI and SI was an excellent predictor of POPF.

**Conclusions:**

It is possible that stapler cartridge selection using our new criteria in combination with CI and SI may reduce the incidence of POPF.

## 1. Introduction

Distal Pancreatectomy (DP) is a procedure for tumors located in the pancreatic body and tail, and is performed for various diseases such as pancreatic cancer, intraductal papillary mucinous tumor, mucous-producing pancreatic tumor, pancreatic solid pseudopapillary neoplasm, pancreatic neuroendocrine tumor, and extra-pancreatic tumors invading to the pancreas. Postoperative pancreatic fistula (POPF) is often experienced after DP, and is closely related to serious complications such as intra-abdominal abscess, sepsis, and pseudoaneurysm formation/rupture.^[1,2]^ Despite the recent dramatical improvement in medical technology, surgical techniques, and perioperative managements, the incidence of POPF after DP remains high, ranging from 10–40%.^[1,3,4]^ For avoiding POPF after DP, several surgical procedures such as hand sutures/main pancreatic duct ligation,^[5,6]^ use of a linear stapler,^[7,8]^ pancreatico-enteric anastomosis,^[9,10]^ use of serous patches,^[11]^ use of fibrin preparations,^[12,13]^ and use of polyethylene glycol mesh,^[14]^ have been reported previously.

In recent years, especially with the rise of laparoscopic and robotic surgery, pancreatic dissection using a linear stapler has been widely performed. Diener et al^[15]^ have shown in a randomized controlled multicenter study that pancreatic resection using a linear stapler shows no effects on reducing the incidence of POPF after DP, when compared with the closure of pancreas cut surface by hand sutures. In contrast, many reports have demonstrated that the incidence of POPF is reduced by the use of automatic sutures.^[2,5,7,8,16]^ Moreover, risk factors influencing the occurrence of POPF after DP using a liner stapler is not fully understood. Therefore, no clear consensus is established for the choice of a liner stapler during DP.

Recently, it has been reported that the choice of the suitable stapler cartridge matching to the thickness of the pancreas is one of the most important factors for preventing the occurrence of POPF. Sugimoto et al^[17]^ have defined staple height/pancreatic thickness as Compression Index (CI) and shown that CI ≤ 0.160 is an independent predictor of POPF. Another study by Nishikawa et al^[18]^ have calculated the difference between pancreatic parenchymal thickness and staple height, and stated that POPF is significantly decreased if the difference is ranged from 6 to 12 mm. However, no previous studies have focused on the relation between staple height and the diameter of main pancreatic duct (MPD).

Therefore, the purpose of this study was to evaluate whether the relation between staple height and pancreatic thickness or MPD diameter influenced the incidence of POPF, and to establish new criteria for stapler cartridge selection during DP.

## 2. Objectives and methods

### 2.1. Objectives

Patients who underwent DP between April 2015 and April 2022 at the Department of Surgery, Saitama Red Cross Hospital, Saitama, Japan, were retrospectively reviewed. Patients receiving combined resection of other organs such as liver, stomach, colon, kidney were excluded from this cohort. Patients receiving major vascular resection and reconstruction were also excluded. Multidetector computed tomography (MDCT) was performed for all patients before surgery.

### 2.2. Methods of DP

Experienced hepato-biliary-pancreatic surgeons participated as primary surgeons or first assistants, and there was no difference in the method of pancreatic dissection between laparotomy and laparoscopy. The pancreas was dissected using either the Echelon® (Johnson & Johnson, New Brunswick) or the Signia® (Medtronic, Minneapolis), which is a 3-row linear stapler. The choice of linear stapler and its cartridges, the use of a preload seat at the time of dissection, and the time required for dissection were dependent on each surgeon. The staple height at the time of closure was determined according to the product information. Precisely, Echelon® cartridges of blue (1.50 mm), gold (1.80 mm), green (2.00 mm), and black (2.30 mm), and Signia® cartridge of purple (1.5–2.25 mm; mean 1.875 mm) were used in this study. In all cases, the line of dissection was determined by a preoperative surgical conference. For most patients with malignant disease, the pancreatic dissection line was just above the portal/superior mesenteric vein, and for benign or low-grade malignant tumors, the dissection line was determined according to the tumor location. If the line of dissection was determined intraoperatively, its location in relation to the major vessels was confirmed and documented in the surgical record. Contextual splenectomy was performed routinely, and lymph node dissection was performed as necessary. One closed suction drain was placed in the vicinity of the pancreatic stump from the right side of the abdomen. The texture of the pancreas was determined by the surgeon’s palpation during surgery in the case of laparotomy or after removal of the pancreatic specimen in the case of laparoscopic surgery.

### 2.3. Perioperative management

The patients were treated with a fast-track protocol, and oral intake was started on postoperative day (POD) 2–8, depending on their condition. Second-generation cephem antibiotics was used during operation and on POD 1. Amylase levels in serum and drainage fluid were measured on POD 1, 3, 5 and 7 and POPF was diagnosed based on the International Study Group of Pancreatic Fistula (ISGPF) 2016 definition.^[19]^ In this study, grade B and C were defined as POPF, and biochemical leakage was excluded from POPF. If there was no POPF, or biochemical leakage and no signs of infection, the drain was removed after POD 3. In cases of prolonged leakage of pancreatic juice or signs of infection in the drainage fluid, drainage was continued and the drain was removed when the fistula was confirmed to be gone.

### 2.4. Definition of Compression Index and Suturing Index

**Figure 1.**
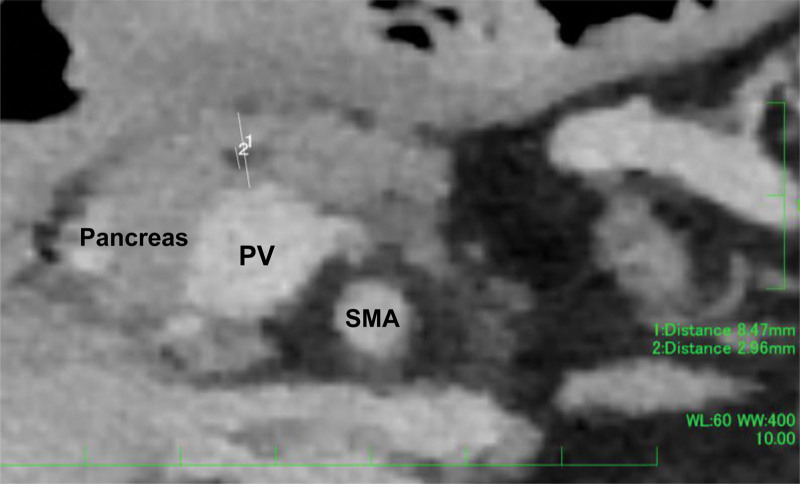
The dissection line during DP was confirmed based on the surgical record, and the thickness of the pancreas and the main pancreatic duct (MPD) diameter at the dissection line were measured using preoperative MDCT. DP = distal pancreatectomy, MDCT = multidetector computed tomography, MPD = main pancreatic duct.

The pancreatic thickness and the MPD diameter at the dissection line were measured using 1-mm-slice high-resolution MDCT axial images taken preoperatively (Fig. 1). In this present retrospective study, these were measured at the site where the pancreas was dissected during DP, referring to surgical records (e.g., just above the superior mesenteric vein or in the right edge of the superior mesenteric artery, and so on). All measurements were performed using the similar protocol by 1 surgeon researcher who was unaware of the POPF results. Compression Index (CI) was defined as the value of staple height divided by pancreatic thickness (staple height/pancreatic thickness), and Suturing Index (SI) was defined as the value of staple height divided by MPD diameter (staple height/MPD diameter).

### 2.5. Statistical analyses

Statistical analyses were performed with the commercially available software EZR version 1.54. All data are expressed as mean ± standard deviation. Statistical comparisons for significance were performed using the Mann–Whitney *U* test, Student *t* test, or Fisher’s exact test. Significant risk factors associated with POPF evaluated by univariate logistic regression were included in a multivariable analysis to determine independent risk factors. The receiver operating characteristic (ROC) analysis was used for the determination of the cutoff value of each variable. Probability (*P*) values of .05 or less were considered statistically significant. Fully informed consent was obtained from all patients. The study was approved by the Ethics Committee for Saitama Red Cross Hospital, Japanese Red Cross Society, Approval No.22-AD, and it conforms to the provisions of the Declaration of Helsinki. Informed consent was obtained in the form of opt-out.

## 3. Results

### 3.1. Risks of POPF in relation to pancreatic thickness or MPD diameter

**Figure 2.**
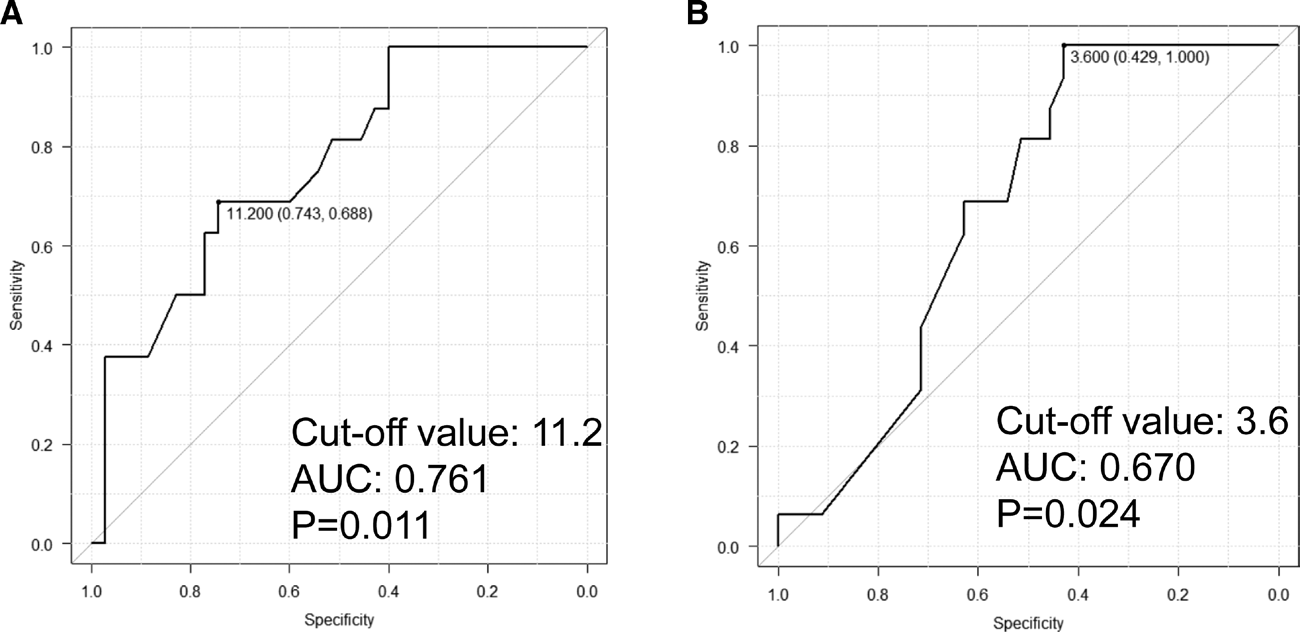
ROC analyses of (A) pancreatic thickness (*P* = .011, AUC = 0.761, cutoff value = 11.2) and (B) MPD diameter (*P* = .024, AUC = 0.670, cutoff value = 3.6) in accordance with the occurrence of POPF. AUC = area under curve, MPD = main pancreatic duct, ROC = receiver operating characteristic.

In this study period, 51 patients underwent distal pancreatectomy without other organ resections. Details of these 51 patients were shown in Table 1. POPF occurred in 16 patients (31.4%). ROC curve analyses revealed that thick pancreatic tissue and small MPD diameter were significant risk factors; and, these AUC were 0.761 and 0.670, respectively (Fig. 2A,B).

**Table 1:** Patient characteristics of 51 patients receiving DP.

Parameters	N = 51
Age (yr)	67.0 ± 13.5
Sex	
Male	23 (45.1%)
Female	28 (54.9%)
BMI (kg/m^2^)	22.7 ± 3.7
Method	
Open surgery	27 (52.9%)
Laparoscopic surgery	24 (47.1%)
Diagnosis	
PDAC	26 (51.0%)
Others	25 (49.0%)
Pancreatic texture	
Hard	6 (11.8%)
Soft	45 (88.2%)
Pancreatic thickness	10.9 ± 3.8
Main pancreatic duct diameter	3.0 ± 1.3
Type of stapler cartridge	
Echelon blue	8 (15.6%)
Echelon gold	2 (3.9%)
Echelon green	10 (19.6%)
Echelon black	19 (37.3%)
Signia purple	12 (23.5%)
POPF	
None or Biochemical leakage	35 (68.6%)
Grade B	15 (29.4%)
Grade C	1 (2.0%)

POPF was defined based on ISGPF.

BMI = body mass index, DP = distal pancreatectomy, ISGPF = International Study Group of Pancreatic Fistula, PDAC = pancreatic ductal carcinoma, POPF = postoperative pancreatic fistula.

### 3.2. Risks of POPF in relation to Compression Index or Suturing Index

**Figure 3.**
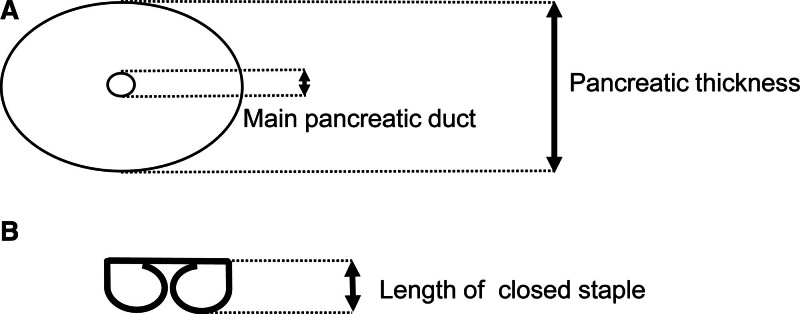
(A) Measurement of pancreatic thickness and MPD diameter using preoperative MDCT. (B) Determination of the length of closed staple according to the product information. MDCT = multidetector computed tomography, MPD = main pancreatic duct.

**Figure 4.**
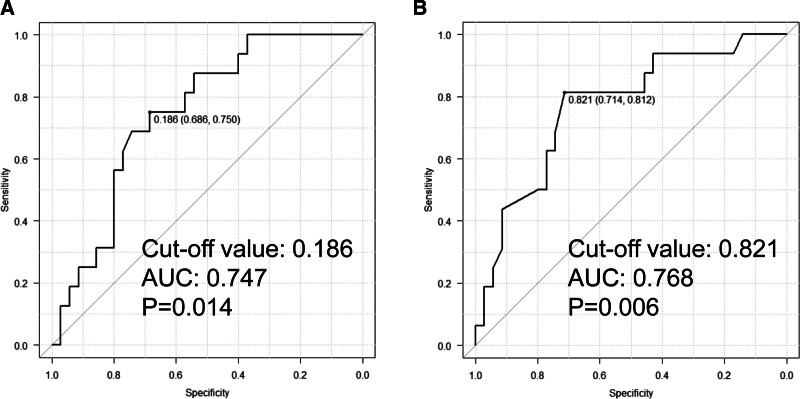
ROC analyses of (A) Compression Index (CI; *P* = .014, AUC = 0.747, cutoff value = 0.186) and (B) Suturing Index (SI; *P* = .006, AUC = 0.768, cutoff value = 0.821) in accordance with the occurrence of POPF. AUC = area under curve, CI = Compression Index, POPF = postoperative pancreatic fistula, ROC = receiver operating characteristic, SI = Suturing Index.

Pancreatic thickness and MPD diameter were measured as described in Figure 3A, using preoperative MDCT. Length of closed staple was also determined as described in Figure 3B, based on the product information. When ROC curve analyses were used to evaluate the adequate cutoff value of CI and SI to differentiate patients with POPF (Fig. 4A,B), significant correlations were found between POPF and CI or SI (*P* = .014 and *P* = .006, respectively), and the adequate cutoff value of CI and SI in accordance with the occurrence of POPF were 0.186 and 0.821, respectively. AUC of CI and SI in accordance with POPF were 0.747 and 0.768, respectively. In contrast to AUC of MPD diameter, AUC of SI was relatively high, especially. These results suggested that adequate stapler selection decreased the occurrence of POPF even in patients with thick pancreas tissue and thin MPD.

### 3.3. Predictors of POPF after DP

**Figure 5.**
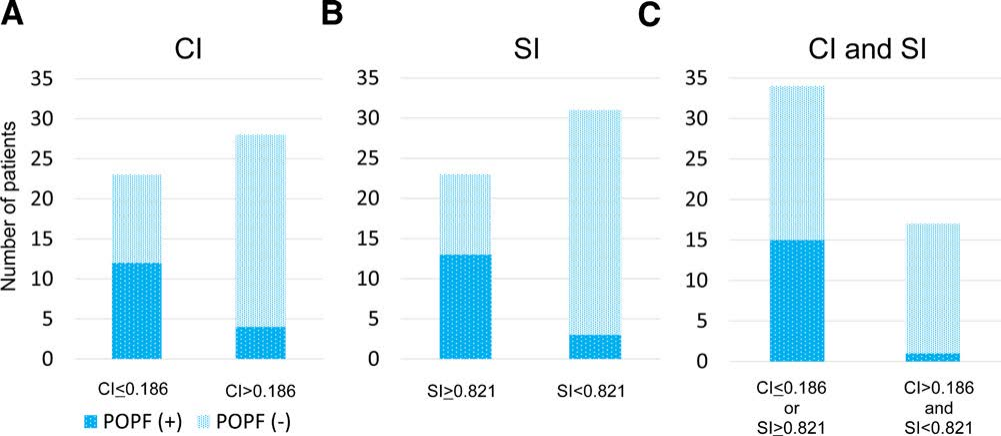
The incidence of POPF based on (A) CI (*P* = .004, POPF false negative rate = 14.3%), (B) SI (*P* < .001, POPF false negative rate = 10.7%), and (C) the combination of CI and SI (*P* < .001, POPF false negative rate = 5.9%). CI = Compression Index, POPF = postoperative pancreatic fistula, SI = Suturing Index.

To evaluate the risks of POPF after DP, comparisons were made between groups with and without POPF (Table 2). Increased operative time and blood loss, thick pancreas tissue, and thin MPD were significant risk factors for POPF. In addition, CI was significantly lower and SI was significantly higher in patients with POPF. To reveal the risk factors of POPF after DP, several variables were analyzed by univariate and multivariate logistic regression models (Table 3). These analyses revealed that CI ≤ 0.186 and SI ≥ 0.821 were independent risk factors for POPF after DP. The incidence of POPF were further investigated based on CI, SI, and the combination of CI and SI. In patients with lower CI (CI ≤ 0.186), 52.2% of patients developed POPF; however, in patients with higher CI (CI > 0.186), only 14.3% of patients developed POPF (Fig. 5A). Moreover, 56.5% of patients with higher SI (SI ≥ 0.821) developed POPF, but only 10.7% of patients with lower SI (SI < 0.821) developed POPF (Fig. 5B). Interestingly, the incidence of POPF in patients fulfilling both CI > 0.186 and SI < 0.821 was only 5.9%, which was extremely lower than in those without fulfilling the criteria (44.1%; Fig. 5C), suggesting that this new criteria in combination with CI and SI was an excellent predictor of POPF after DP.

**Table 2:** Clinicopathological features of patients with or without POPF.

	POPF	None or BL	*P* value
	(n = 16)	(n = 35)	
Age (yr)	72.0 ± 7.9	64.7 ± 14.9	.086
Sex (male/female)	10/6	13/22	.096
Body weight (kg)	61.8 ± 12.2	56.9 ± 9.4	.138
Height (cm)	162.3 ± 8.7	159.3 ± 7.3	.205
BMI (kg/m^2^)	23.4 ± 3.9	22.5 ± 3.5	.408
Albumin (g/dL)	4.1 ± 0.3	4.1 ± 0.4	.436
Amylase (IU/I)	80.0 ± 34.5	94.3 ± 67.1	.427
HbA1c (%)	6.4 ± 1.3	6.3 ± 1.1	.821
Operative time (min)	328.9 ± 94.3	272.6 ± 67.5	**.039**
Blood loss (g)	693.4 ± 631.1	276.3 ± 386.3	**.017**
Surgical procedure (open or laparoscopic)	9/7	18/17	.749
PDAC (yes or no)	9/7	17/18	.611
Pancreatic texture (hard or soft)	2/14	4/31	.912
Pancreatic thickness (mm)	13.2 ± 3.7	9.9 ± 3.4	**.011**
Main pancreatic duct diameter (mm)	2.3 ± 0.6	3.3 ± 1.4	**.024**
Echelon blue (length of closed staple: 1.5 mm)	1	7	.236
Echelon gold (length of closed staple: 1.8 mm)	1	1	.572
Echelon green (length of closed staple: 2.0 mm)	3	7	.917
Echelon black (length of closed staple: 2.3 mm)	7	12	.518
Signia purple (length of closed staple: 1.875 mm)	4	8	.867
Compression Index	0.165 ± 0.037	0.218 ± 0.072	**.014**
Suturing Index	0.939 ± 0.251	0.676 ± 0.275	**.006**

POPF was defined based on ISGPF. *P* < 0.05 are indicated in bold to emphasize the significance.

BL = biochemical leakage, BMI = body mass index, ISGPF = International Study Group of Pancreatic Fistula, PDAC = pancreatic ductal carcinoma, POPF = postoperative pancreatic fistula.

**Table 3:** Logistic regression analysis of prognostic factors associated with POPF.

Variable	Univariate analysis		Multivariate analysis Model 1		Multivariate analysis Model 2	
	Odds ratio	*P* value	Odds ratio	*P* value	Odds ratio	*P* value
Age ≥57 yr	6.87 (0.80–58.80)	.078				
Male sex	2.82 (0.83–9.58)	.096				
Body weight > 61.7 kg	3.11 (0.86–11.30)	.084				
Height > 162.5 cm	2.46 (0.74–8.26)	.144				
BMI > 20.7 kg/m^2^	1.99 (0.47–8.42)	.352				
Albumin < 4.0 g/dL	0.67 (0.20–2.19)	.504				
Amylase > 67 IU/I	0.40 (0.12–1.36)	.142				
HbA1c < 5.8%	1.57 (0.47–5.28)	.462				
Operative time > 302 min	5.14 (1.42–18.70)	**.013**	6.26 (1.11–35.40)	**.038**	6.19 (0.87–44.10)	.069
Blood loss > 440 g	4.00 (1.11–14.40)	**.034**	6.68 (1.08–41.50)	**.042**	17.9 (1.42–226.0)	**.026**
Laparoscopic surgery	0.82 (0.25–2.71)	.749				
PDAC	1.36 (0.41–4.47)	.611				
Soft pancreas	1.11 (0.18–6.77)	.912				
Pancreatic thickness > 11.2 mm	4.81 (1.36–17.10)	**.015**	7.97 (1.37–46.50)	**.021**	Not included	
Main pancreatic duct diameter < 3.6 mm	11.2 (1.33–94.90)	**.026**	21.0 (1.83–241.0)	**.014**	Not included	
Echelon blue (length of closed staple: 1.5 mm)	0.27 (0.03–2.38)	.236				
Echelon gold (length of closed staple: 1.8 mm)	2.27 (0.13–38.70)	.572				
Echelon green (length of closed staple: 2.0 mm)	0.92 (0.21–4.15)	.917				
Echelon black (length of closed staple: 2.3 mm)	1.49 (0.45–5.00)	.518				
Signia purple (length of closed staple: 1.875 mm)	1.12 (0.28–4.47)	.867				
Compression Index ≤ 0.186	6.55 (1.72–24.90)	**.006**	Not included		32.8 (2.53–426.0)	**.008**
Suturing Index ≥ 0.821	37.00 (2.75–498.0)	**.006**	Not included		15.9 (2.27–112.0)	**.005**

*P* < 0.05 are indicated in bold to emphasize the significance.

BMI = body mass index, PDAC = pancreatic ductal carcinoma.

## 4. Discussion

POPF is one of the most serious complications after DP. Various reports have previously been reported regarding the risk factors of POPF after DP, such as age,^[5,20,21]^ sex,^[22,23]^ body mass index (BMI),^[5,20,21,23,24]^ operative time,^[8,9,24]^ blood loss,^[24]^ pancreatic thickness,^[7,20,23,25]^ texture of pancreas,^[25,26]^ extended lymph node resection,^[22]^ additional organ resection,^[5]^ and pancreatic resection method. Recent studies, De Pastena et al^[27]^ reported an increased risk of POPF while increasing the pancreatic thickness and the MPD diameter. For closure of pancreas cut edge, 2 common methods, closure of the pancreas by hand sutures and dissection by a linear stapler, are recognized as standard. With the progress of laparoscopic and robotic surgery, closure by linear stapler is increasing in particular. However, there is still no consensus on the optimal choice of stapler cartridge for DP, and there are only a few reports discussing the relationship between stapler cartridge and POPF.

Sugimoto et al^[17]^ have defined staple height/pancreatic thickness as CI and shown that Compression Index ≤ 0.160 was an independent predictor of POPF. These data suggest that POPF is more likely to occur at lower staple heights. In the present study, lower CI was also an independent risk factor for POPF, and its cutoff was 0.177. Our data was similar to the value evaluated by Sugimoto, et al. In addition, we defined staple height/MPD diameter as SI in our study and evaluated the usefulness of SI for predicting POPF. We clearly showed that SI ≥ 0.821 was an independent risk factor for POPF; however, these results were against the fact that POPF increased in lower CI with lower staple height. In general, the thickness of the target tissue is an issue when dissecting tissue with a linear stapler. As for cases with CI ≤ 0.186, a low staple height relative to the pancreatic substance increases the possibility of damage to the pancreatic capsule and pulverization of the pancreatic parenchyma, resulting in pancreatic juice leakage from the pancreatic parenchyma. Other reports have also discussed the occurrence of POPF in relation to pancreatic thickness and stapler cartridge. Kim et al^[28]^ have defined the pancreatic thickness/staple height as the compression ratio (CR) and showed that a high CR is an independent risk factor for POPF. However, they did not determine the adequate cutoff value of CR. They have also compared the incidence of POPF by dividing patients into 3 groups based on the pancreatic parenchymal thickness, and revealed that POPF tends to increase as the pancreatic parenchyma became thicker.

In contrast, in patients with SI ≥ 0.821, it seems that the high staple height causes insufficient closure of MPD, leading to the leakage of pancreatic juice from MPD. In fact, MPD tended to be contrasted by the agent injected from drain during drain exchange, when SI was 1.00 or higher (*P* = .017), suggesting that insufficient closure of MPD in patients with higher SI is a trigger for the occurrence of POPF. Nishikawa et al^[18]^ have defined the difference between pancreatic parenchymal thickness and staple height as height difference (HD), and found that POPF is significantly decreased in patients with HD ranged from 6 to 12 mm. They have stated that pancreatic juice leaks through the gap of the closed staple in cases with staple height higher than the necessary height, because of the insufficient compression of MPD. This finding is similar to our results of SI which reveals that excess levels of high height staple is unable to close MPD completely.

Therefore, it is necessary to close both the pancreatic parenchyma and the MPD with a staple for preventing POPF. For achieving these conflicting methods, appropriate stapler should be selected which fulfill the condition of CI ≤ 0.186 and SI ≥ 0.821. Surprisingly, the incidence of POPF in the group of patients fulfilling both CI ≤ 0.186 and SI ≥ 0.821 was remarkably low, as it was only 5.9%. For selecting appropriate staple cartridge, the site of pancreatic dissection should be predicted preoperatively, and thickness of the pancreatic parenchyma and the diameter of MPD at the site of dissection should be measured using MDCT, which resulting in the reduced risks of POPF.

There are several limitations in the present study that need to discuss. First, this study was conducted retrospectively at a single institution, and the sample size of patients was small. Second, DP combined resection of other organs were excluded from this study, as the POPF rate was very high in DP cases with resection of other organs. The reason of increased POPF in patients with combined resection of other organs might be a prolonged operation time with a large amount of blood loss, suggesting that incidence of POPF increases as surgical stress increases. Third, the choice of stapler cartridge was determined intraoperatively by each surgeon, and there was no fixed method for closing the anvil jaws of the stapler cartridge. Recently some reports have described the usefulness of prolonged compression before dividing the pancreas to avoid POPF.^[29,30]^ Fourth, the method of measuring pancreatic thickness and MPD diameter is controversial. Pecorelli et al^[31]^ reported that correlation of preoperative radiological pancreatic neck diameters with pathology measures of the dissection margin were valid, but the results of our study, measured by a single surgeon researcher, must be considered with skepticism. In addition, there have been recent reports^[32]^ showing the effectiveness of stapler cartridges using polyglycolic acid (PGA), and it is possible that the use of these methods in this study could have further reduced the incidence of POPF. Considering the above, to confirm the usefulness of our new criteria determined by the combination of CI and SI, randomized controlled multicenter trial should be conducted.

## 5. Conclusions

In conclusion, CI ≤ 0.186 and SI ≥ 0.821 may be good predictors of POPF risk after DP in this study. It is possible that stapler cartridge selection using our new criteria in combination with CI and SI may reduce the incidence of POPF, but further study is necessary to investigate how to reduce the incidence of POPF.

## Author contributions

**Conceptualization:** Kei Naito.

**Data curation:** Kei Naito.

**Formal analysis:** Kei Naito.

**Methodology:** Kei Naito.

**Supervision:** Kazuyasu Shinmura, Takayuki Suzuki, Shintaro Maeda, Satoshi Kuboki, Masayuki Ohtsuka.

**Validation:** Kei Naito.

**Writing – original draft:** Kei Naito.

**Writing – review & editing:** Kei Naito.
